# Utilization of ^134^Cs/^137^Cs in the environment to identify the reactor units that caused atmospheric releases during the Fukushima Daiichi accident

**DOI:** 10.1038/srep31376

**Published:** 2016-08-22

**Authors:** Masamichi Chino, Hiroaki Terada, Haruyasu Nagai, Genki Katata, Satoshi Mikami, Tatsuo Torii, Kimiaki Saito, Yukiyasu Nishizawa

**Affiliations:** 1 Sector of Nuclear Science Research, Japan Atomic Energy Agency, 2-4 Shirakata, Tokai-mura, Naka-gun, Ibaraki 319-1195, Japan; 2 Sector of Fukushima Research and Development, Japan Atomic Energy Agency, 7-1 Taira Omachi, Iwaki, Fukushima 970-8026, Japan; 3 Sector of Fukushima Research and Development, Japan Atomic Energy Agency, 2-2-2 Uchisaiwai-cho, Chiyoda-ku, Tokyo, 100-8577, Japan; 4 Sector of Fukushima Research and Development, Japan Atomic Energy Agency, 45-169 Harama-chiku Kaibama sukakeba, Minamisoma City, Fukushima 975-0036, Japan

## Abstract

The Fukushima Daiichi nuclear power reactor units that generated large amounts of airborne discharges during the period of March 12–21, 2011 were identified individually by analyzing the combination of measured ^134^Cs/^137^Cs depositions on ground surfaces and atmospheric transport and deposition simulations. Because the values of ^134^Cs/^137^Cs are different in reactor units owing to fuel burnup differences, the ^134^Cs/^137^Cs ratio measured in the environment was used to determine which reactor unit ultimately contaminated a specific area. Atmospheric dispersion model simulations were used for predicting specific areas contaminated by each dominant release. Finally, by comparing the results from both sources, the specific reactor units that yielded the most dominant atmospheric release quantities could be determined. The major source reactor units were Unit 1 in the afternoon of March 12, 2011, Unit 2 during the period from the late night of March 14 to the morning of March 15, 2011. These results corresponded to those assumed in our previous source term estimation studies. Furthermore, new findings suggested that the major source reactors from the evening of March 15, 2011 were Units 2 and 3 and that the dominant source reactor on March 20, 2011 temporally changed from Unit 3 to Unit 2.

Since 2011, we have been estimating the source term—temporal changes in atmospheric release rates (Bq/h) of radionuclides—caused by the Fukushima Daiichi nuclear power station (FDNPS) accident using a reverse estimation method that combines atmospheric dispersion simulation and environmental monitoring data^1^,^2^,^3^,^4^,^5^. Many international researchers have also tried the source term estimation and model simulation of atmospheric dispersion of radionuclides during the accident. The United Nations Scientific Committee on the Effects of Atomic Radiation (UNSCEAR) summarized sixteen results on source term estimation (Table B2 of UNSCEAR 2013 Report^6^). It described that the source term estimated by Terada *et al.*^3^ (which is the one from our previous study) provided a sound basis for estimation of the levels of radioactive material in the terrestrial environment where prior measurements did not exist and actually the dispersion and deposition of released material modeled by the World Meteorological Organization (WMO) based on the source term by Terada *et al.*^3^ could replicate the broad pattern of deposition density of ^137^Cs over the Japanese land mass. We also summarized a number of international papers lately that have carried out the source term estimation and numerical analysis of atmospheric dispersion process of radionuclides released during the accidents (Table 1 of Katata *et al.*^5^).

The accuracy of our previous study’s latest source term increased with gradual increases in the number of monitoring data after the accident and improvement of our team’s numerical simulation model that included a sophisticated atmospheric deposition scheme^5^. The calculated ground-shine due to the large deposition event of March 15–16, 2011 agreed with observed data within a factor of 2 at most of the monitoring points, and the model also reproduced the spatial distribution of the airborne survey’s air dose rate and ^137^Cs surface deposition within a factor of 5. Therefore, the simulation results of the spatiotemporal patterns of ^137^Cs surface deposition have enough accuracy to compare with the observed ^134^Cs/^137^Cs ratio, though some discrepancies between simulation and observation occurred because of model simulation uncertainties. Using the latest source term in Katata *et al.*^5^, several atmospheric dispersion simulations by the National Oceanic and Atmospheric Administration (NOAA), USA, Canadian Meteorological Centre (CMC), and Met Office, UK, were successfully able to reproduce the measured surface contamination distribution and time series in air concentrations of radionuclides regardless of model structure and meteorological input data^5^. UNSCEAR also reported for Katata *et al.*^5^ that in any further or updated assessment, the committee would recommend the use of the latest estimate as “preferential”^7^.

While the timing and quantities of major atmospheric releases during the FDNPS accident had been estimated, the relationships between these releases and their specifically correlated reactor units have still not been clarified. During the period of March 12–15, 2011, the temporal rises in air-dose rates measured by a monitoring car at the FDNPS boundary were partially connected to the events that occurred in the reactors^8^. However, after March 15, 2011, although only a few studies investigated the potential reasons why the atmospheric releases continued for such a long period afterward^9^,^10^, the precise rationale behind the event still has not been verified definitively.

Therefore, this paper focuses on the reactor units that generated large ^137^Cs atmospheric releases during the period of March 12–21, 2011.

## General Methodology

The employed method supporting this evaluation was “reverse identification” via the use of environmental monitoring data and the atmospheric transport and deposition model, the Worldwide version of SPEEDI (WSPEEDI-II).

It is known that the ^134^Cs/^137^Cs inventory ratio in a reactor core varies depending on the extent of fuel burnup, indicating that each FDNPS reactor has its own distinctive ratio value. Here, fuel burnup is essentially a measure of how much energy is extracted from nuclear fuel, which is usually expressed as energy released per unit-mass of initial fuel. By assuming that the ^134^Cs/^137^Cs ratio for each reactor was consistently maintained during the discharge pathway to the atmosphere, atmospheric transport, surface deposition, and migration within soil, the measured ^134^Cs/^137^Cs ratio for ground-deposition could be used to specify the areas respectively contaminated from Unit 1, 2, and/or 3 releases. Meanwhile, WSPEEDI-II simulations were made for each individual time-segment with major releases based on the latest source term estimations to specify the areas contaminated by the major releases^5^. Via a subsequent “area-comparison evaluation,” the reactor units that contributed to associated major atmospheric releases could then be determined.

For determining ^134^Cs/^137^Cs ground surface measurement results, two kinds of datasets were used: (1) *in-situ* gamma spectrometry readings on the land over East Japan^11^ and (2) autonomous unmanned helicopter (AUH) survey readings within a 3-km area of FDNPS^12^.

**Figure 1:**
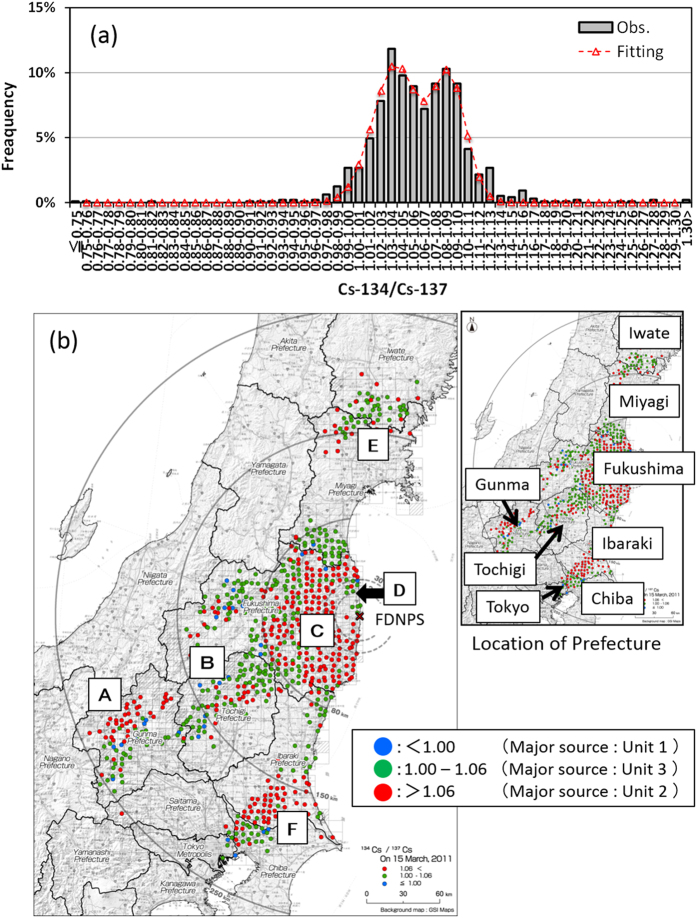
Gamma-ray spectrometry measurement results in the environment (*in-situ* gamma spectrometry), (**a**) histogram of measured ratios of ^134^Cs/^137^Cs (grey boxes) over the measuring points and (**b**) distribution map of the ratio of ^134^Cs/^137^Cs. A dashed line with triangles in (**a**) shows the fitting curve for the measured ratio of ^134^Cs/^137^Cs to a composition of two Gaussian functions by the least squares method. Distribution map of the ratio on ^134^Cs/^137^Cs is created using ArcGIS version 10.2.1, (http://www.esri.com/) with administrative boundary data and background map by The Geospatial Information Authority of Japan (http://maps.gsi.go.jp/).

Figure 1 shows the *in-situ* gamma spectrometry measurement results: (a) the frequency distribution of measured ^134^Cs/^137^Cs ratios (decay-corrected to March 15, 2011) at every measurement point and (b) its spatial distribution on the land over East Japan. The frequency distribution contains two peaks: one appears at the ^134^Cs/^137^Cs value of 1.09, which is almost the same as the inventory of Unit 2 (1.08) estimated by Nishihara *et al.*^13^, and the other of 1.04 is very close to the inventory of Unit 3 (1.05). The minimum between the two peaks appears at the value of 1.06. In Fig. 1b, the 715 individual ^134^Cs/^137^Cs ratio points where the value of ^137^Cs surface deposition was greater than 10 kBq/m^2^ are collectively plotted as the spatial distribution. Here, red circles depict the ^134^Cs/^137^Cs ratio > 1.06 (corresponding to Unit 2 releases); green circles depict the ^134^Cs/^137^Cs ratio between 1.00 and 1.06 (corresponding to Unit 3 releases); and blue circles depict the ^134^Cs/^137^Cs ratio < 1.00 (corresponding to Unit 1 releases). As shown in Fig. 1a, the value of 1.06 is “median-prone” between the peak values for Units 2 and 3, and the value of 1.00 is “median-prone” for Units 1 and 3. According to the latter, the Unit 1 peak value (0.95) is discussed in greater detail below. Figure 1b, moreover, represents the following characteristics via spatial distributions of the areas contaminated by releases from Units 2 and 3:

(1) The area of red circles (Unit 2) distributed around the western mountainous area in the Gunma Prefecture (Area A);

(2) The area of green circles (Unit 3) around the Gunma, Tochigi and the south of Fukushima Prefectures (Area B);

(3) The area of red circles (Unit 2) at the eastern part of the Fukushima Prefecture (Area C), which also include green circles (Unit 3) in the northwest direction of FPNDS (Area D);

(4) The area of green circles (Unit 3) around the boundary of Iwate and the Miyagi Prefectures (Area E); and

(5) The area of red circles (Unit 2) at the southern part of the Ibaraki Prefecture (Area F).

**Figure 2:**
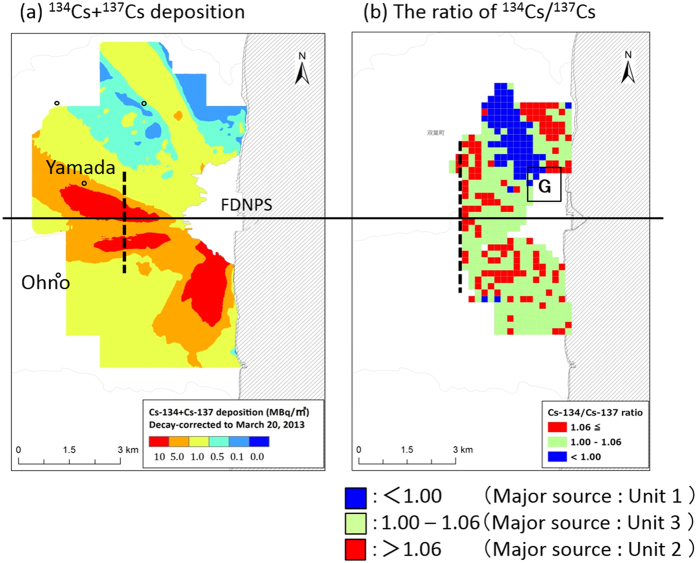
Autonomous unmanned helicopter (AUH) survey; spatial maps of (**a**) ^134^Cs+^137^Cs deposition over the 5-km area of FDNPS^27^ and (**b**) the ^134^Cs/^137^Cs ratio over the 3-km area. The spatial distribution maps are created using ArcGIS version 10.3 (http://www.esri.com/) with administrative boundary data and background map by The Geospatial Information Authority of Japan (http://maps.gsi.go.jp/).

Figure 2 presents the overall measurement results of the AUH survey, which consisted of the following: (a) the spatial distribution in ^134^Cs+^137^Cs depositions over the 5-km area around FDNPS, and (b) the ^134^Cs/^137^Cs ratio (decay-corrected to March 15, 2011) over the 3-km area. In Fig. 2b, the spatial distribution of the ^134^Cs/^137^Cs ratios is shown as the same color chart used in Fig. 1b. Here, the ^134^Cs/^137^Cs ratios for 200-m grid square are median values for each grid square in which one hundred measured data were obtained on average. The average ratios of ^134^Cs/^137^Cs in four high-deposition areas shown in Fig. 2a, e.g., the north-northwest, upper-west, lower-west, and south, are 0.95, 1.06, 1.05, and 1.05, respectively. The ^134^Cs/^137^Cs ratio (0.95) in the north-northeast area is virtually the same as the inventory of Unit 1 (0.94). However, because the AUH survey does not portray enough accuracy to clearly distinguish the areas specifically influenced by Unit 2 versus Unit 3 (as discussed in detail within the Materials and Methods section below), it is therefore assumed that the areas of the upper-west, lower- west, and south were specifically affected by Units 2 and 3. Hence, the associated local contamination distribution characteristics around FNDPS were deemed as the following:

(1) The area of blue blocks (Unit 1) toward the north-northwest direction (Area G), and

(2) The area of red and green blocks (Units 2 and 3) from the west to the south.

Uncertainty of both measurements, *in-situ* gamma spectrometry measurement and the AUH survey, is discussed in the Materials and Methods section.

**Figure 3:**
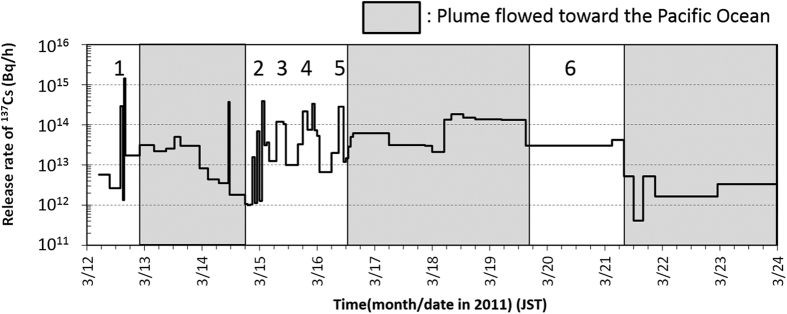
Temporal variation of atmospheric release rate of ^137^Cs estimated by Katata *et al*.^5^. The number means the major releases which contaminated the land mass of East Japan.

Numerical simulations on atmospheric dispersion and deposition of radionuclides were carried out by the Worldwide version of SPEEDI (WSPEEDI-II)^14^ with a new deposition scheme via Katata *et al.*^5^. The temporal change in release rate of ^137^Cs from March 12–24, 2011 estimated by Katata *et al.*^5^ is shown in Fig. 3. The three dark-shaded time periods depict when the plume mainly flowed toward the Pacific Ocean, which resulted in a temporary absence of cesium ground deposition data. The figure shows that relatively large release quantities continued until almost March 21, 2011 and that six distinct instances of major releases had contaminated the soil surface. Thus, the WSPEEDI-II simulations for the following release periods were carried out to identify the deposition areas created by the subject major releases:

- Release periods from 15:30 Japanese Standard Time (JST) to 16:00 JST on March 12, 2011 (***Case 1***).
- From 21:00 JST on March 14, 2011 to 02:00 JST on March 15, 2011 (***Case 2***).
- From 07:00 to 11:00 JST on March 15, 2011 (***Case 3***)
- From 16:00 JST on March 15, 2011 to 01:00 JST on March 16, 2011 (***Case 4***).
- From 09:00 to 11:00 JST on March 16, 2011 (***Case 5***), and
- From 00:00 JST on March 20 to 06:00 JST on March 21, 2011 (***Case 6***).

## Results

**Figure 4:**
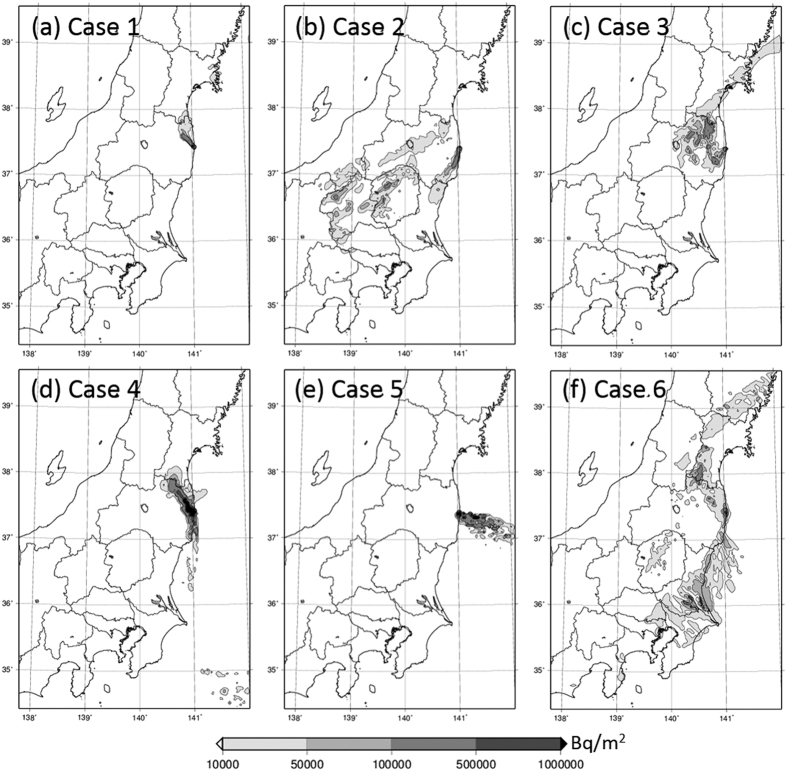
Deposition patterns of ^137^Cs over East Japan calculated by WSPEEDI-II for the six cases when large amounts of releases occurred. Depositions for *Case 1* (**a**), *Case 2* (**b**) to *Case 5* (**e**), and *Case 6* (**f**) are temporally accumulated until 14:00 JST on March 13, 2011, 00:00 JST on March 17, 2011, and 00:00 JST on March 24, 2011, respectively. The spatial distribution maps are created using GMT (Generic Mapping Tools)^28^ Version 4.5.2 (http://gmt.soest.hawaii.edu/) with administrative boundary data by the Geospatial Information Authority of Japan (http://maps.gsi.go.jp/).

**Figure 5:**
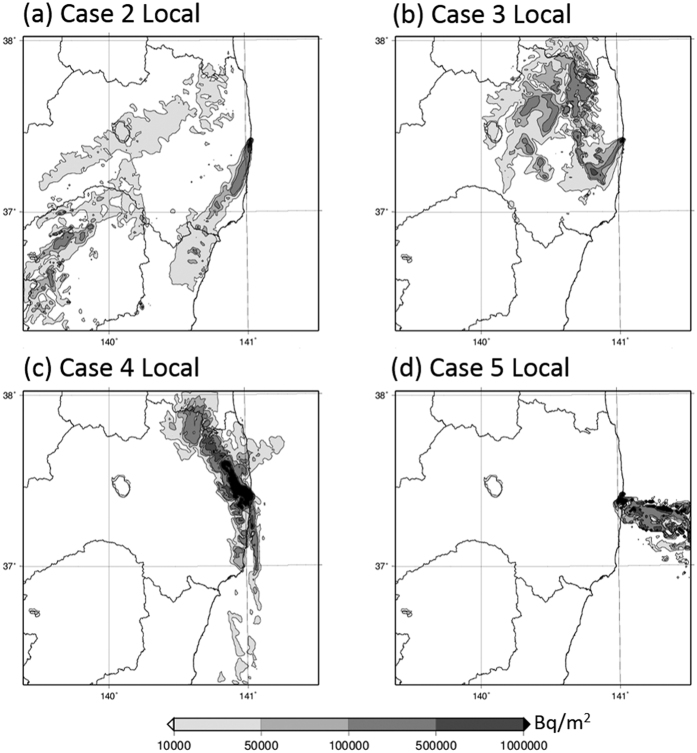
Deposition patterns of ^137^Cs accumulated until 00:00 JST on March 17, 2011 over the Fukushima Prefecture calculated by WSPEEDI-II for *Cases 2* to *5.* The spatial distribution maps are created using GMT (Generic Mapping Tools)^28^ Version 4.5.2 (http://gmt.soest.hawaii.edu/) with administrative boundary data by the Geospatial Information Authority of Japan (http://maps.gsi.go.jp/).

**Figure 6:**
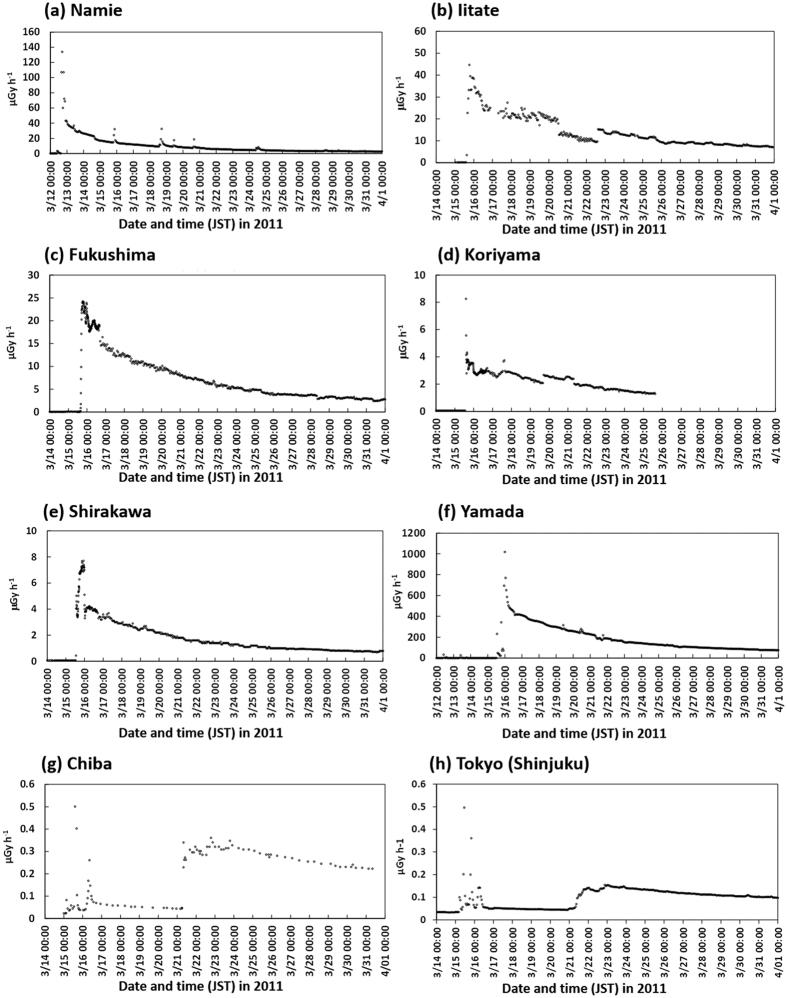
Temporal changes in the air dose rates observed in the Fukushima Prefecture (a^15^, b^16^, c^17^, d^17^, e^17^, f^15^), the Chiba Prefecture (g^18^) and Tokyo-Shinjuku (h^19^).

Deposition patterns of ^137^Cs over East Japan calculated by WSPEEDI-II for the six subject cases are shown in Fig. 4. The deposition patterns of ^137^Cs for Cases 2 to 5 over the Fukushima Prefecture are also shown in Fig. 5. Temporal variations of observed air dose rates at several points over East Japan^15^,^16^,^17^,^18^,^19^ are provided in Fig. 6 for discussion. Hereafter, the described reactor events that occurred in Units 1 through 3 are referenced from Tokyo Electric Power Company Inc. (TEPCO)^8^.

### Case 1 (from 15:30 to 16:00 JST on March 12, 2011)

During the release period of *Case 1*, the hydrogen explosion of Unit 1 occurred at 15:36 JST. Figure 4a for *Case 1* shows that the plume flowed toward the north-northwest (NNW) direction, and ultimately formed a contamination contour (via dry deposition) along the northeastern coast of the Fukushima Prefecture. The plume then changed its direction toward the north-northeast (NNE) direction, resulting in a virtual zero-trajectory for measurement on the land over East Japan (Fig. 1b). However, Fig. 2b clearly shows the narrow band of Unit 1’s ^134^Cs/^137^Cs ratio toward the NNW (Area G), which corresponds to the flow direction of the simulated plume from the hydrogen explosion. After the hydrogen explosion, three monitoring posts of the Fukushima Prefecture^15^ (Shinzan [3.9 km NNW from FDNPS], Namie [8.6 km NNW], and Kiyohashi [8.2 km north]) located at the downwind region of the subject narrow band showed increases of air dose rates at around 17:00 JST on March 12, 2011. Continuous monitoring data for air dose rates at Namie until the end of March 2011 showed that the maximum increase in ground-shine occurred during *Case 1*, with no further drastic increase (in ground-shine) observed after the night of March 12, 2011; however, some peaks did appear due to cloud-shine (Fig. 6a). These results essentially indicated that the blue band in Fig. 2b corresponding to the Unit 1’s ^134^Cs/^137^Cs ratio was created by large amounts of releases associated with the Unit1’s hydrogen explosion.

### Case 2 (from 21:00 JST on March 14, 2011 to 02:00 JST on March 15, 2011)

During the release period of *Case 2*, the safety relief valve (SRV) of Unit 2’s reactor pressure vessel (RPV) was opened at 21:20 JST, followed by respective decreases of RPV pressure at 21:20 and 23:00 JST on March 14, 2011, and at 01:10 JST on March 15, 2011. According to Tanabe^20^ and Katata *et al.*^5^, the coincidence of the timing of air dose rate increases in the environment with the decreases of RPV pressure indicates that atmospheric releases very likely occurred from Unit 2 per the actuation of the SRV.

In the WSPEEDI-II simulation (Figs 4b and 5a), the plume flowed toward the south to south-southwest (SSW) direction and formed a high-contamination-area contour (by dry deposition) along the southeastern coast of the Fukushima Prefecture. The plume then moved westward and encountered a rain-band at the mountainous regions of the Gunma and Tochigi Prefectures and the central part of the Fukushima Prefecture, resulting in a large amount of wet deposition at those locations.

Hot spots at the western part of the Gunma Prefecture coincide with the areas of Unit 2’s ^134^Cs/^137^Cs ratio (Area A in Fig. 1b), suggesting that a large release quantity during the night of March 14, 2011 was, in fact, from Unit 2. The deposition at the central area of the Tochigi Prefecture also appeared through both simulation and observation, though the observation data (in Fig. 1b) does not seem quite as clear as compared to the simulation data. These results hence support the investigations made by Tanabe^20^ and Katata *et al.*^5^.

In Fig. 1b, deposition corresponding to Unit 3 is seen in the area around the Gunma, Tochigi and the south of Fukushima Prefectures (Area B in Fig. 1b). According to Fig. 4, *Case 2* and *Case 6* affected the objective area. Although it is not included in Fig. 4, the WSPEEDI-II simulation for the plume discharged during the period between *Case 2* and Case *3 (Case 2–3)* shows the deposition on the similar area. According to the air dose rates observed at Utsunomiya City^19^, the capital of the Tochigi Prefecture, the large increase appeared on March 15 which corresponds to *Case 2* and *Case 2–3* and the small on March 20–21 to *Case 6*. Because the dominant source of *Case 2* is Unit 2, the deposition corresponding to Unit 3 (Area B) was likely due to a release that occurred in *Case 2*–*3*. The area of green circles at the west part of the Fukushima Prefecture in Fig. 1b might be also created in this period. However, as the WSPEEDI-II simulation could not reproduce the deposition in this area, it is not verified.

### Case 3 (from 07:00 to 11:00 JST on March 12, 2011)

In *Case 3*, the drywell (D/W) pressure of Unit 2 decreased between 07:20 and 11:25 JST. This decrease corresponded to the extremely high air dose rate observed at the main gate from 07:00 to 10:00 JST, indicating a very large release quantity into the atmosphere from Unit 2^20^.

According to the WSPEEDI-II simulation, the plume flowed widely over the eastern part of the Fukushima Prefecture (Fig. 5b). The plume first flowed toward the southwest (SW) direction and reached the central area of the Fukushima Prefecture, including Shirakawa City (81 km WSW) and Koriyama City (58 km W), and then gradually drifted northward and encountered the rain band. During this process, first, the dry deposition stage created a contamination zone over the SW area close to FDNPS in the morning on March 15, 2011; second, wet deposition occurred in the central area of the Fukushima Prefecture around noontime of that day; and lastly, wet deposition also occurred in the NW area of FDNPS including Fukushima City (67.2 km NW) and Iitate-mura (38.9 km NW) in the early evening of that day. The process of the plume first stretching from east to west and then subsequently heading up to Fukushima City and Iitate-mura is confirmed by the fact that increases in air dose rates measured by portable monitors at both locations started at almost exactly the same time (i.e., around 16:00 JST^16^,^17^), regardless of the appreciable distance (28 km) which exists between Fukushima City and Iitate-mura. Continuous monitoring data at Fukushima City, Koriyama City, Shirakawa City, and Iitate-mura until the end of March 2011 show that the maximum increases in ground-shine occurred during *Case 3*, with no further extreme increases detected before/after the afternoon of March 15, 2011 (Fig. 6b–e). It is noted that the NW area close to FDNPS, approximately within 15–20 km, was not contaminated by this plume due to the complex movement of the plume and the lack of a precipitation event.

The heterogeneous contamination distribution shown in Fig. 5b is similar to the area of Unit 2’s ^134^Cs/^137^Cs ratio in the eastern part of the Fukushima Prefecture (Area C in Fig. 1b), suggesting that a large portion of *Case 3’s* release was from Unit 2.

### Case 4 (from 16:00 JST on March 15, 2011 to 01:00 JST on March 16, 2011)

In the WSPEEDI-II simulation, the subject plume first flowed to the west-northwest (WNW) direction, and then moved in a clockwise direction to the northwest (NW) during the evening of March 15, 2011. Around 21:00 JST, via a rapid counterclockwise change of wind direction, the flow returned toward the west around midnight and then finally toward the south during the early morning of March 16, 2011. During this process, a contamination zone was first created in the NW direction in the evening, then in the west area close to the site over the nighttime hours, and finally in the south area of the site over the early morning hours (Fig. 5c). It is noted that although the aggregate contamination level was quantitatively high, the overall impacted area was limited within approximately 50 km to the NW and several km to the west and south from FDNPS. This simulation result is essentially consistent with the fact that the second increase of measured air dose rate at Iitate-mura appeared from 21:00 to 22:00 JST, while a small second peak at Fukushima City at 23:00 JST and successively large increases were observed at Yamada (4.1 km WNW) at 23:00 JST on March 15, 2011 to 00:00 JST on March 16, 2011 and Ohno (4.9 km WSW) at 00:00–01:00 JST on March 16, 2011. The continuous monitoring data at Yamada until the end of March 2011 showed that the maximum increase of ground-shine occurred during *Case 4*, with no further increases being observed before and after the night of March 15, 2011 (Fig. 6f). Thus, the high-deposition zone observed to the west of FDNPS (shown in Fig. 2a) would have been created when the air dose rates at Yamada and Ohno increased during the night of March 15, 2011.

The contamination to the NW direction in Fig. 5c coincides with the narrow band of Unit 3’s ^134^Cs/^137^Cs ratio extended to the NW direction (Area D in Fig. 1b). The green circle arm appeared in observation is actually unclear compared with Fig. 5c due to possible overestimation of deposition to the NW direction of FDNPS in simulation and limited number of observation points within 20 km. Nevertheless, green circle area appeared only in the NW direction within about 50 km (Fig. 1b), suggesting the release mainly from Unit 3 in the evening of March 15.

Furthermore, the ratio of ^134^Cs/^137^Cs in the surrounding area of FDNPS in the west direction in Fig. 2b indicates that the contamination occurred due to atmospheric releases from Units 2 and 3. Thus, the dominant source in the first half of *Case 4*, i.e., when the plume was transported to the NW direction of FDNPS in the evening of the day, would be Unit 3 and the latter half when the plume was transported in the west and south areas close to the site Units 2 and 3.

### Case 5 (from 09:00 to 11:00 JST on March 16, 2011)

During this period, a decrease of D/W pressure was reported at Unit 3 from 09:00 to 11:00 JST on March 16, 2011. In addition, white smoke from the Unit 3 building was observed at 08:30 JST.

The WSPEEDI-II simulation shows that the plume released during the decrease of D/W pressure flowed toward the Pacific Ocean in the morning, returned to the coastal area around noon, and then reversed back toward the Pacific Ocean once again before finally moving deeply into inland areas. This plume movement likely made the dry deposition zone in the west to south directions close to the site (Fig. 5d). However, because the area was also contaminated by the plumes of *Case 2* and *Case 4*, the determination of the source reactor Unit from the ^134^Cs/^137^Cs ratio is difficult. Concerning air dose rates, large increases of such rates to 33 and 324 μGy/h were observed at Matsudate (14.2 km SSW) and Ohno at 11:00 and 12:00 JST on March 16, 2011, respectively. The times of increasing air dose rates at these two locations essentially coincided with the movement of the plume simulated by WSPEEDI-II. This result hence indicates that the decline in Unit 3’s D/W pressure between 09:00 and 11:00 JST accompanied a large quantity of associated releases, and consequently, prompted a substantial increase in air dose rates within the environment.

### Case 6 (from 00:00 JST on March 20, 2011 to 06:00 JST on March 21, 2011)

According to the movement of the subject plume simulated by WSPEEDI-II, it was estimated that while extending southward it had subsequently migrated to a landfall position from the Pacific Ocean during the morning of March 20, 2011 and had accordingly formed low deposition areas on the northeastern part of the Ibaraki Prefecture and on the entire Tochigi Prefecture. Next, the plume headed north toward the Miyagi Prefecture. Finally, it flowed southward again and formed a large deposition zone over the southern part of the Ibaraki Prefecture, the northern part of the Chiba Prefecture and the eastern part of Tokyo (Fig. 4f). Continuous monitoring data at Tokyo-Sinjuku (225 km SSW) and Chiba City (211 km S) through the end of March 2011 show that the maximum increase of ground-shine occurred during *Case 6*, with no further increases via ground-shine being observed before/after the morning of March 21, 2011. Some peaks, however, due to cloud-shine appeared during March 15–16, 2011 (Fig. 6g,h).

Via comparison with Fig. 1b, the deposition areas in the Tochigi Prefecture and at the boundary between the Miyagi and Iwate Prefectures were likely caused by the release from Unit 3 (Area E in Fig. 1b), and the deposition over the southern part of the Ibaraki Prefecture was likely caused by the release from Unit 2 and partially from Unit 3 (Area F in Fig. 1b). Therefore, the dominant source reactor changed from Unit 3 to Unit 2 at a certain time during the period of *Case 6*.

## Discussion

The major source reactor units for *Cases 1* to *3* have been investigated in some prior studies, mainly through analyzing the timing between events in the reactors and the increases of air dose rates around FDNPS^5^. This present study hence validates such past investigations via the use of a new, independent method. This also means that the method described in this subject study can be universally effective for identifying responsible source reactor Units after a nuclear accident event. In the case of the Fukushima disaster, new results indicate that the “major release” sources were likely Units 2 and 3 during *Case 4* and that the dominant source reactor Unit changed, with time, for *Case 6*. Thus, additional focus for discussion is placed upon these two cases in detail below.

*Case 4* includes two events in the reactors which potentially caused atmospheric releases. The first event was the increase in air dose rate measured by the Unit 2 containment atmosphere monitoring system (CAMS) at 15:25 JST, following the decrease of D/W pressure between 13:00 and 15:50 JST on March 15, 2011. The maximum value on the CAMS appeared at 16:10 JST and gradually decreased with time, accompanying the gentle decrease of D/W pressure. Here, the increase of air dose rates per the CAMS implies that radioactive gas had filled the containment vessel and the continuous decrease of values on CAMS and D/W pressure was correlated with the ongoing atmospheric release of such gases from the vessel.

The second event was the venting operation started from 16:05 JST at Unit 3 and the decline in D/W pressure between 16:00 JST on March 15, 2011 and 02:05 JST on March 16, 2011. These occurrences imply the possibility of releases from both Units 2 and 3. The result in *Case 4* essentially indicates that the event at Unit 3 probably caused a larger quantity of release during the evening time and that the events at Units 2 and 3 together likely caused releases during the nighttime period. This determination is hence further examined below from an environmental monitoring data perspective.

**Figure 7:**
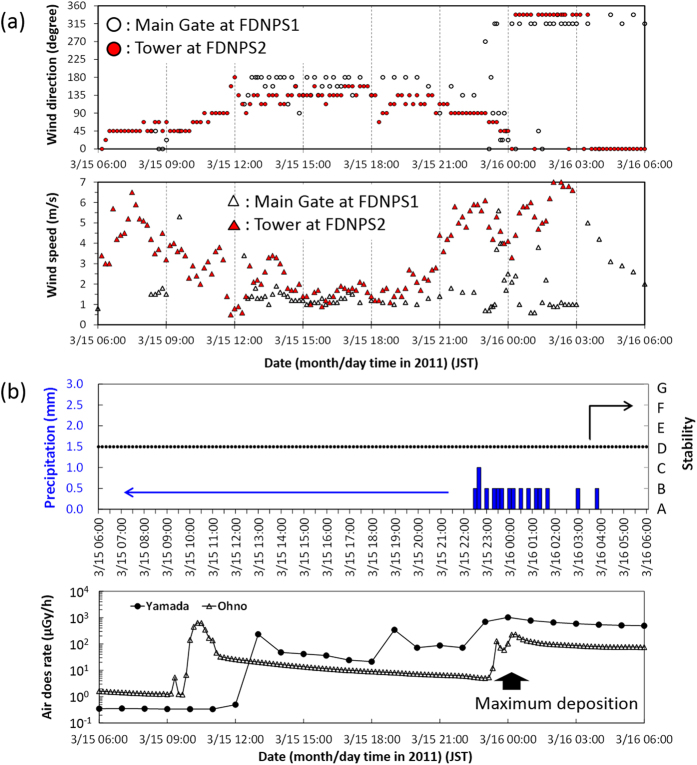
Meteorological and radiation monitoring data around the site; (**a**) the meteorological data measured at the tower of FDNPS-2 whose height is 120 m above ground level and those at the ground level at the main gate of FDNPS; (**b**) the temporal variation of precipitation amount and atmospheric stability at the Ohno monitoring post and air dose rates at two monitoring posts, Yamada and Ohno. The temporal resolution of available data at Ohno was every 10 min., while Yamada every hour.

The temporal variations to meteorological and air dose rate data on March 15, 2011 are shown in Fig. 7, which essentially consisted of the following: (a) varying meteorological data measured at the FDNPS-2 tower whose height is 120 m above ground-level^21^ compared to those at ground-level (the FDNPS main gate^22^), and (b) the temporal variation of air dose rates at two monitoring posts at Yamada (4.1 km WNW) and Ohno (4.9 km WSW) and precipitation amount and atmospheric stability at Ohno^23^. In Fig. 7b, the air dose rate measured at Yamada increased during 18:00–19:00 JST. At this time, the wind direction at the tower of FDNPS-2 changed from SE to ENE at 18:20 JST, although the wind direction at the ground level measured at the FDNPS main gate remained southward. If an elevated release occurred during the period of 18:00–19:00 JST, the plume would firstly approach Ohno, and then change the direction to Yamada before reaching Ohno, and finally pass through Yamada around 19:00 JST. Hence, the increase in air dose rate at Yamada at 18:00–19:00 JST and the decrease at 19:00–20:00 JST was likely therefore due to the elevated release.

At midnight of March 15, 2011, as mentioned in *Case 4*, the high deposition area appeared in the west direction close to FDNPS as shown in Fig. 2a. In the figure, the high deposition zone started from about 2 km downwind. This distribution pattern is “normally” made by dry deposition of a plume released from an elevated source. The downwind distances where the maximum deposition appeared are approximately 3.8 km for the upper west area and 2.7 km for the lower west area. It is consistent that an elevated release of 120 m is likely to result in a peak dry deposition at 2–5 km downwind under the neutral atmospheric condition. Small precipitation amounts were recorded at Ohno intermittently around midnight. However, if the high deposition area was really created mainly by dry deposition due to the discharge of radionuclides during an interval of precipitation, the deposition pattern would also show that an elevated release occurred.

It is reasonable to therefore consider that these elevated releases likely occurred via the Unit 3 venting episodes. However, as the possibility of plume rise from the Unit 2 reactor building and/or Unit 3 also remains, further investigation is necessitated to definitively determine the reactor event(s) that caused major releases.

**Figure 8:**
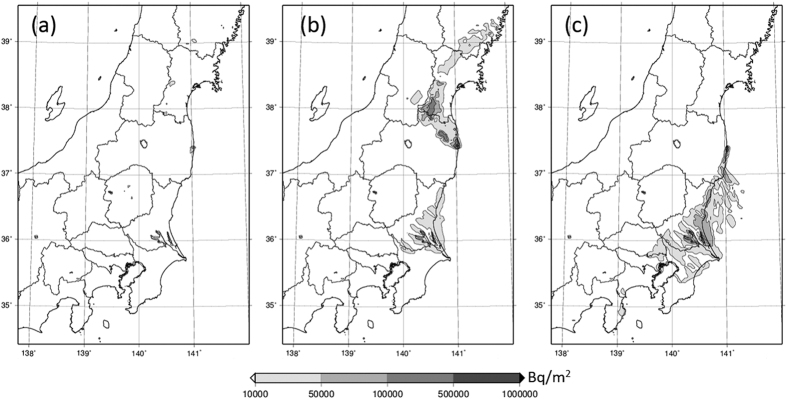
Deposition distributions of ^137^Cs accumulated until 00:00 JST on March 24, 2011 over East Japan for three subdivided release periods in *Case 6.* The spatial distribution maps are created using GMT (Generic Mapping Tools)^28^ Version 4.5.2 (http://gmt.soest.hawaii.edu/) with administrative boundary data by the Geospatial Information Authority of Japan (http://maps.gsi.go.jp/).

Concerning *Case 6*, the major source reactor changed over time. The timing of the change is characterized by dividing the release period of *Case 6* into individual 10-hours increments; i.e., the first release at 00:00 to 10:00 JST on March 20, 2011, the second at 10:00 to 20:00 JST on March 20, 2011, and the third at 20:00 JST on March 20, 2011 to 06:00 JST on March 21, 2011. The source term is then re-estimated for the three individuated release cases. According to Katata *et al.*^5^, the release rates of ^137^Cs in *Case 6* are 3.0 × 10^13^ Bq/h during the period from 00:00 JST on March 20, 2011 to 03:00 JST on March 21, 2011 and 4.2 × 10^13^ Bq/h from 03:00 to 06:00 JST on March 21, 2011. The former was an averaged value of release rates estimated from five separate dust-sampling data sets obtained at different times and places. When we estimate release rates from the five data sets separately, the former can be divided into relatively low release rates on the order of 10^12^ Bq/h from two data obtained before around 11:00 JST and higher release rates on the order of 10^13^ Bq/h from three data obtained after 11:00 JST (Table 1). By averaging the release rates obtained from each data for the three aforementioned subdivided periods, re-estimated ^137^Cs release rate for each release period can hence be determined, as shown in Table 1. Using these new release rates, the deposition distributions of ^137^Cs in East Japan for the three subject release periods calculated by WSPEEDI-II are shown in Fig. 8a–c.

The WSPEEDI-II simulation for the first release shows that the plume discharged during this period initially flowed toward the south, migrated inland from the Pacific Ocean, crossed the east coast of Ibaraki, and finally reached the central area of East Japan from the Tochigi to the Iwate Prefectures. However, due to relatively low release rates, the deposition zone for the first release remains unclear (Fig. 8a), compared with the second and third releases.

During the second release, the plume meandered toward the NW direction and then reached the boundary of the Miyagi and Iwate Prefectures (Fig. 8b). During this period, a venting operation was carried out at Unit 3 from 11:25 JST on March 20, 2011 until the decrease of D/W pressure (which continued until 10:00 JST on March 21, 2011) indicated that the atmospheric release had occurred. In fact, the ratio of ^134^Cs/^137^Cs (Area E in Fig. 1b) shows that the contamination zone at the boundary of the Miyagi and Iwate Prefectures should be due to the Unit 3 release. It is noted that the decline in D/W pressure continued until 10:00 JST on March 21, 2011. If a large quantity of releases from Unit 3 continued over the entire term of the pressure decrease, it would also have resulted in contamination at the southern part of the Ibaraki Prefecture, which was probably affected by the Unit 2 plume (Area F in Fig. 1b). The reasons were not altogether clarified or fully comprehended, but the release rate from Unit 3 apparently decreased (significantly) around the end of this subject period.

According to the WSPEEDI-II simulation for the third release, the plume flowed toward the south. During this period, the major contamination area was at the south portion of the Ibaraki Prefecture via wet deposition, with Fig. 1b illustrating that the contamination was due to the release from Unit 2 (Area F in Fig. 1b). The reactor data of Unit 2, in which the D/W pressure was almost the same as the atmospheric pressure, implies that the releases from Unit 2 continued through the time of *Case 6* and became dominant when the releases from Unit 3 (due to venting and/or other possible events) were terminated. Another possibility, however, is that Unit 2 ^137^Cs releases ultimately increased due to some other unknown mechanism. From around 20:00 JST on March 20, 2011, there were no reactor data signals available.

This paper described the approach of combining the measurement of ^134^Cs/^137^Cs on ground surfaces and atmospheric transport/deposition simulations that could identify which reactor discharge large radionuclide quantities from accidental releases that can occur from multiple-reactor facilities like the Fukushima Daiichi nuclear station. Katata *et al.*^5^ just speculated about the connection between major release events estimated from environmental data and the accident events reported by TEPCO^8^. The present study is novel on the point that we analyzed the new data of ^134^Cs/^137^Cs ratio and reasonably identified the source units that created high dose rate areas.

However, in many cases, it could not be directly determined which event occurrence in a particular reactor ultimately resulted in a large quantity of releases. Thus, as a future endeavor to improve the overall safety and reliability of commercial nuclear power systems, the events which caused the major releases during the FPNDS accident should be revealed by integrated research efforts using a series of this team’s study findings, analyses of various reactor and environmental data, and severe reactor accident simulations.

As shown in Table 1, the release rates of ^137^Cs for the period from 00:00 JST on March 20 to 06:00 JST on March 21, 2011 were re-estimated. The source term on the temporal variation in release rates of ^137^Cs during March 2011 can be updated by dividing the value from 15:00 JST on March 19 to 08:00 JST on March 21 in Supplement (141115_Suppl_acp_JAEAsourceterm.csv) of Katata *et al.*^5^ into three values of 7.0 × 10^12^, 4.6 × 10^13^ and 4.3 × 10^13^ Bq/h for 15:00 JST on March 19 - 10:00 JST on March 20, 10:00 JST - 20:00 JST on March 20, and 20:00 JST on March 20 - 08:00 JST on March 21, respectively. Although the release amount in the period was slightly decreased from our previous results^5^, this did not cause a significant change in the total amount of release of ^137^Cs for March 11 to 31, 2011 (14.1 PBq in this study).

## Material and Methods

### Inventories in Units 1 to 3

Nishihara *et al.*^13^ analyzed inventories in Units 1 to 3 by using ORIGEN2 code^23^. The ORIGEN2 code calculates weight, radioactivity, heat generation, photon generation and neutron generation rate of each radionuclide for 688 activation products, 128 actinides, and 879 fission products. On the basis of the result from Nishihara *et al.*^13^, the ^134^Cs/^137^Cs ratios of inventories in Units 1 to 3 at the shutdown time (March 11, 2011) are estimated as the highest for Unit 2 (1.08), the lowest for Unit 1 (0.94), and intermediate value for Unit 3 (1.05).

### *In-situ* measurement

*In-situ* gamma spectrometry was conducted from December 2011 to May 2012 to obtain the deposition density of radioactivity deposited on the land over East Japan, under contract with the Ministry of Education, Culture, Sports, Science and Technology (MEXT). Details of observational methods and results are described in Mikami *et al.*^11^. It is considered that systematic errors in absolute detection efficiency for a Germanium (Ge) detector can be canceled by taking the ratio of ^134^Cs to ^137^Cs because the gamma-ray detection efficiency is similar for ^134^Cs (605 keV) and ^137^Cs (662 keV). In fact, the uncertainty accompanying the ^134^Cs/^137^Cs ratio associated with intrinsic random nature of radioactivity was estimated to be approximately 0.6% from the counts of photopeaks at 605 keV and 662 keV, respectively. In addition, the variation of the ratio among several different Ge detectors observed for an intercomparison carried out exactly at the same place was generally 1–2%. Komori *et al.*^24^ has reported the ^134^Cs/^137^Cs ratio at 55 separate locations in the Fukushima, Kanto area, etc. via soil sample analysis. Direct comparisons between identical locations are difficult because their sampling locations were not the same as those utilized for our study. However, part of the data provided in the Komori *et al.*^24^ showed that the ^134^Cs/^137^Cs ratio obtained in the Kanto region was 1.00–1.04 (on March 11, 2011). These findings are comparable with this study’s results for those obtained in nearly the same geographic area.

The frequency distribution of measured ^134^Cs/^137^Cs ratios (Fig. 1a) is not well described with a single Gaussian function, but rather well fitted by the least-squares method to depict a double-Gaussian function. One peak appears at the ^134^Cs/^137^Cs value of 1.09, which is almost the same as the inventory of Unit 2 (1.08) estimated by Nishihara *et al.*^13^, with another at 1.04 that is close to Unit 3 (1.05); the minimum between the two peaks appears at the value of 1.064. Here, there is no scientific reason that the distribution is Gaussian, but it was nonetheless employed for fitting as a trial. The fact that no peak appears at the ^134^Cs/^137^Cs value of 0.95 means that the cesium releases from Unit 1 did not significantly contribute to the deposition on the land over East Japan. In addition, considering that the two peaks appeared close to 1.05 and 1.08, and a bimodal Gaussian fitting is applicable, the areas where the effects of releases from Units 2 and 3 appeared comparable are probably not so large. The reason that the deposition areas affected by the releases from Units 2 and 3 on the land over East Japan could be separated is that the time-integrated meteorological conditions for long-term travel of each radioactive plume were multifarious, even if the released directions of some plumes were the same close to the source. Here, a small number of blue circles randomly appeared on the land over East Japan do not show the contribution from Unit 1. The Gaussian distribution of the ^134^Cs/^137^Cs ratios of 1.00–1.06 has the small number of data with smaller ratios than 1.00 (these represents as blue circles) by the statistically minor probability in the tails of the Gaussian distribution. Therefore, the blue circles are supposed to be appearing in green-circle region of A, B, and F. The slight differences between the peak values in the environment and the ^134^Cs/^137^Cs inventory ratios in Units 2 and 3 were likely caused by the reason that the inventory ratios were calculated from the total inventories of ^134^Cs and ^137^Cs, while the ratios measured in the environment somewhat depended on the melted areas in the reactor core at release time. The reason why the ratio of ^134^Cs/^137^Cs in the environment maintains a distribution is probably due to the variation of core melting area over time, as well as to the “multi-source effect.” The frequency distribution assessment mentioned above indicates that bimodal distributions with two peaks in Fig. 1a represent the ratio of ^134^Cs/^137^Cs discharged from Units 2 and 3, respectively. Since bimodal frequency distributions of the ^134^Cs/^137^Cs ratio in Fig. 1a overlap one another, the areas affected by Unit 2 and 3 releases cannot be separated completely using the certain value of the ^134^Cs/^137^Cs ratio (i.e., 1.06). However, because the areas of red (Unit 2) and green circles (Unit 3) in Fig. 1b distribute with boundaries, this value is in fact enough to roughly distinguish the areas contaminated by Unit 2 and Unit 3. Here, if the deposition occurred at the same location resulting from two or more units but the magnitude of the deposition was significantly larger for one of the units, the ratio of ^134^Cs/^137^Cs resulting from the “smaller release” will be masked and not evident on Fig. 1b. Thus, this method can identify only dominant source, even if the releases occurred from two or more units.

### Unmanned helicopter survey

An autonomous unmanned helicopter (AUH) was used to measure the detailed radioactive cesium distribution within a 3-km area of FDNPS under the commission of the Nuclear Regulatory Agency. Because the AUH was equipped with a LaBr_3_(Ce) scintillation detector, which detects a high-resolution spectrum of gamma-rays compared to the spectrum of NaI detector systems typically employed for aerial monitoring, peak distinctions of ^134^Cs (796 and 802 keV) and ^137^Cs (662 keV) are possible. The ^134^Cs/^137^Cs ratios were taken from measurement data obtained during August-October 2012. Associated details of observational methods and data analysis are described in Nishizawa *et al.*^12^. Concerning the uncertainty of the ^134^Cs/^137^Cs ratio, in the areas where the total deposition of ^134^Cs + ^137^Cs exceeded 1 MBq/m^2^, i.e., the north-northwest, upper west, lower west and south, Interquartile Range (IQR) in 200-m grid squares was within 0.2, which corresponded to the standard error of approximately 1–2%. According to the comparison in the ^134^Cs/^137^Cs ratio between AUH measurements and *in-situ* measurements using a Ge detector at eight points within a 3 km area of FDNPS, the differences averaged about 2%^12^. The accuracy of AUH measurement is probably lower than *in-situ* measurement, due to the error included in estimating the deposition levels on the ground from the peak energy of radioactive cesium measured at an average altitude of 80 m above the ground. Thus, although the average ratios of ^134^Cs/^137^Cs in four high-deposition areas shown in Fig. 2a; i.e., the north-northwest, upper west, lower west and south are 0.95, 1.06, 1.05, and 1.05, respectively^12^, our study team determined it was best not to employ the data from the AUH survey for attempting to discern contributions between Units 2 and 3, which required a high degree of accuracy. Its use was ultimately employed, however, to identify the deposition area generated by Unit 1 whose ^134^Cs/^137^Cs ratio value was apparently lower than those of Units 2 and 3. One interpretation for green area is the sum effect from Unit 1 and Unit 2. However, the fact that the averaged values of the ^134^Cs/^137^Cs ratio in the upper west, lower west and south direction from FDNPS are close to inventories of Units 2 and 3 implies that such a sum effect was very small.

### Numerical simulation

**Figure 9:**
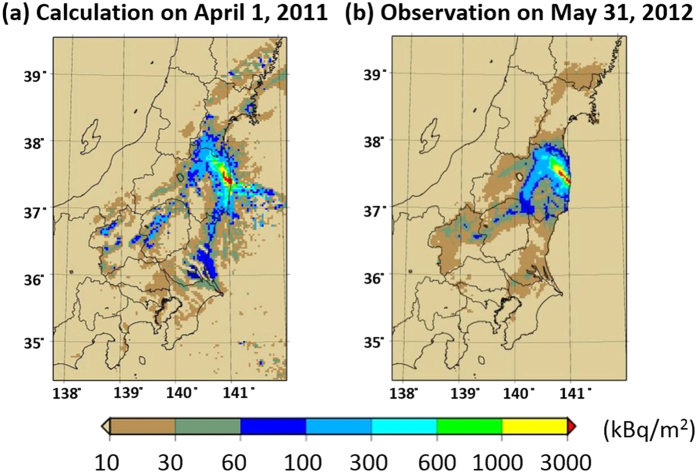
Comparison of the spatial distributions of surface deposition of ^137^Cs over East Japan by (**a**) the simulation and (**b**) observation (from Figure 13 (**d,e**) of Katata *et al.*^5^). This work is licensed under the Creative Commons Attribution 3.0 Unported License. To view a copy of this license, visit http://creativecommons.org/licenses/by/3.0/ or send a letter to Creative Commons, PO Box 1866, Mountain View, CA 94042, USA. The spatial distribution maps are created using GMT (Generic Mapping Tools)^28^ Version 4.5.2 (http://gmt.soest.hawaii.edu/) with administrative boundary data by the Geospatial Information Authority of Japan (http://maps.gsi.go.jp/).

The worldwide version of SPEEDI (WSPEEDI-II) consists of a non-hydrostatic mesoscale atmospheric model MM5^25^ and a Lagrangian particle dispersion model GEARN^26^. Both models must have the sophisticated capability to address dry and wet depositions of radionuclides for comparing predicted deposition areas with observed ones. Per this aspect, MM5 has many options for parameterizations of cloud micro-physics, cumulus clouds, planetary boundary layers (PBLs), radiation, and land surface schemes. By using meteorological fields predicted by MM5, GEARN calculates the atmospheric dispersion of radionuclides by tracing the trajectories of a large number (typically a million) of marker particles discharged from a release point. Some portion of the airborne radioactivity is then deposited on the ground surface by turbulence (dry deposition) and precipitation (wet deposition). GEARN has been modified to use a sophisticated deposition scheme, which entails dry and fog-water deposition, cloud condensation nuclei activation, and subsequent wet scavenging due to mixed-phase cloud microphysics (in-cloud and below-cloud scavenging) for radioactive iodine gas (I_2_ and CH_3_I) and other particles (CsI, Cs, and Te)^5^. The spatial deposition pattern of ^137^Cs in East Japan calculated by WSPEEDI-II throughout March 2011 corresponded to measured values^5^, indicating that WSPEEDI-II with the latest source term is available for the purpose of this study (Fig. 9).

The input data on temporal variation of release rates and release heights for each period are obtained from Katata *et al.*^5^. The atmospheric dispersion simulations by GEARN were carried out until almost all marker particles flowed out of the computational domain. The computational domain, spatial resolutions, meteorological data, geographical data, and other model parameters are the same as those utilized in Katata *et al.*^5^.

## Additional Information

**How to cite this article**: Chino, M. *et al.* Utilization of ^134^Cs/^137^Cs in the environment to identify the reactor units that caused atmospheric releases during the Fukushima Daiichi accident. *Sci. Rep.* **6**, 31376; doi: 10.1038/srep31376 (2016).

## Figures and Tables

**Table 1:** Re-estimation of release rates of ^137^Cs for three subdivided periods of *Case 6.*

Sampling location*	Sampling date and time* (JST)	Observed ^137^Cs Conc.* (Bq m^−3^)	Calculated ^137^Cs Conc.* (Bq m^−3^)	Release rate of ^137^Cs (Bq h^−1^)	Released date and time of plume	Release rate for each period (Bq h^−1^)
JAEA	20/3 11:35	26	4.7 × 10^−12^	5.5 × 10^12^	20/3	First period: 7.0 × 10^12^
Tokai	−20/3 11:55				04 JST	
MEXT	20/3 11:37	970 (^131^I)	2.2 × 10^−11^	8.5 × 10^12^	20/3	
41**	−20/3 11:49		(^131^I)		08 JST	
MEXT	20/3 14:13	1000	1.5 × 10^−11^	6.7 × 10^13^	20/03	Second: 4.6 × 10^13^
21	−20/3 14:33				11 JST	
MEXT	20/3 14:15	180	1.1 × 10^−11^	1.6 × 10^13^	20/3	
31	−20/3 14:35				11 JST	
MEXT	20/3 14:45	4100	1.3 × 10^−11^	6.1 × 10^13^	20/3	
46**	−20/3 14:55	(^131^I)	(^131^I)		11 JST	
JAEA	21/3 03:45	438	1.1 × 10^−11^	4.0 × 10^13^	20/03	Third: 4.0 × 10^13^
Tokai	−21/3 07:05				22 JST	

^*^After Katata *et al.*^5^

^**^At MEXT41 and 46, only data on ^131^I were available; thus, the release rates of ^137^Cs were calculated by estimating the release rates of ^131^I and multiplying the ratio of ^137^Cs/^131^I observed at other points at the similar time.
